# 

**Published:** 1900-03-03

**Authors:** 


### NOTICE TO CORRESPONDENTS.

All MS., Letters, Books for Review, and other matters intended for the Editor should be addressed The Editor, "The Hospital," The Hospital Building, 28 & 29, Southampton Street, Strand, W.C.

The Editor cannot undertake to return rejected MS., even when accompanied by stamped directed envelope.

**Notice to Advertisers and Subscribers.**

All Advertisements, Orders for Copies of The Hospital, and Business Communications should be addressed to **THE MANAGER (Not to the Editor)**, The Hospital, The Hospital Building, 28 & 29, Southampton Street, Strand, London, W.C.

The Cost of Subscription, post free, is as follows:—Per week, 2½d. per quarter, 2s. 8d.; per half-year, 5s. 3d.; per annum, 10s. 6d. Foreign:—Per annum, 15s. 2d., payable, in all cases, in advance.

**NOTICE.—Vol. XXVI. of "The Hospital" (April, 1899, to September, 1899), in handsome cloth binding, is now on sale at the Office of this Journal, price 7s. 6d. Cases for binding the Numbers contained in this Volume 1s. 6d. Index to Vol. XXVI., 2½d., post free.**

**The Monthly Part for March is now ready. Price 6d., by post 8d.**

### The Student of Medicine.

### APPOINTMENTS.

J. Battersby, F.R.C.S., appointed Surgeon to the Central Dispensary, Glasgow. R. A. Bayliss, M.R.C.S.Eng., L.R.C.P., Lond., appointed Honorary Medical Officer to the Eastern Dispensary, Bath. H. J. Bell, L.R.C.P., L.R.C.S.-Edin., appointed Medical Officer for the Mortlake District of the Richmond Union. F. C. Bottomley, M.D., B.C., B.A.Camb., M.R.C.S., L.R.C.P., appointed Assistant Surgeon to the Boscombe Hospital. W. W. Chipman, M.B., C.M., F.R.C.S.Edin., appointed Assistant Gynæcologist to the Royal Victoria Hospital, Montreal. S. A. Clarke, L.R.C.P.I., L.M., L.S.A., appointed District Medical Officer of the West Ham Union. W. H. Coltart, M.R.C.S., L.R.C.P., appointed House Physician to Brompton Hospital for Consumption and Diseases of Chest, London, S.W. R. F. Craggs, M.D. Durh., appointed Ophthalmic Surgeon to the Children's Hospital, Newcastle-on-Tyne. C. C. Douglas, M.D., B.Sc.Edin., F.F.P.S.Glasg., appointed Pathologist to the Glasgow Maternity Hospital. A. M. Erskine, M.B., B.Ch.Irel., D.P.H., appointed an Honorary Surgeon to the Goole Cottage Hospital. W. Hallam, M.R.C.S.Eng., L.S.A., appointed Surgeon to the Brightside Division of the Sheffield Police. C. C. Hayward, M.B., B.S., M.R.C.S., L.R.C.P., appointed House Physician to Brompton Hospital for Consumption and Diseases of the Chest, London, S.W. H. C. Lawrence, M.D., L.R.C.P.Lond., M.R.C.S., appointed Medical Officer to the Male Department of the Cheltenham Training College. J. D. E. Mortimer, M.B., F.R.C.S., appointed Registrar and Anæsthetist (pro tem.) to the Royal Hospital for Women and Children, Waterloo Bridge Road. H. S. Pendlebury, F.R.C.S., appointed Assistant Surgeon to the Royal Hospital for Children and Women, Waterloo Bridge Road, S.E. R. I. Richardson, M.B., C.M.Edin., appointed Visiting Physician to the Liverpool City Hospital North, Netherfield Road. W. S. Richardson, M.D.Brux., L.R.C.P.Lond., M.R.C.S., appointed Assistant Physician to the Boscombe Hospital, Bournemouth. G. A. Rorie, M.B., B.S.Edin., appointed Junior Assistant Medical Officer to the Cumberland County Council Asylum. J. S. E. Selby, B.A.Camb., M.R.C.S., L.R.C.P.Lond., appointed Medical Officer for the Seventh District of the Aylesbury Union. S. B. Stedman, M.R.C.S., L.R.C.P.Lond., appointed Medical Officer for the Binbrook District of the Louth Union. F. J. Steward, M.S., F.R.C.S., appointed Assistant Surgeon to Guy's Hospital; also Assistant Surgeon to the Hospital for Sick Children, Great Ormond Street.

### BOOKS FOR NURSES.

Domville's Manual for Hospital Nurses, 8th edition ... ... ... ... ... ... ... 2/6

Cullingworth's Monthly Nursing, 4th edition... 1/6

Hellier's Gynæcological Nursing... ... ... 1/6

Cuff's Lectures on Medicine to Nurses .. ... 3/6

Richmond's Antiseptic Principles for Nurses 1/-

Mayne's Short Dictionary of Medical Terms, 2/6

Chavasse's Advice to a Wife, 14th edition, by Dr. Fancourt Barnes ... ... ... ... 2/6

Chavasse's Advice to a Mother, 15th edition, by Dr. George Carpenter .. ... ... ... 2/6

London: J. & A. Churchill, 7, Great Marlborough Street.

### NOW READY,

A REPRINT OF

### A NURSING HANDBOOK

TOGETHER WITH S. MAW, SON, AND THOMPSON'S

### COMPLETE CATALOGUE OF NURSING REQUISITES.

The above work is now republished at a cost of 1s. per copy. Messrs. S. M., S., and T. have been compelled to make this charge on account of the extreme popularity of the work and the great expense incurred in its production.

***Post Free on receipt of 1s.***

Nurses' Bags.

### S. MAW, SON, and THOMPSON,

7 to 12, ALDERSGATE STREET, LONDON, E.C.

